# Urban-rural differences in China’s crude death rate changes

**DOI:** 10.1186/s12889-022-12717-9

**Published:** 2022-02-25

**Authors:** Fan Xiao, Li Mei, Quanbao Jiang

**Affiliations:** Institute for Population and Development Studies, School of Public Policy and Administration, Xi’an Jiaotong University, No.28 Xianning West Road, Xi’an, Shaanxi China

**Keywords:** Crude death rate, Age-specific death rate, Age structure, Decomposition, Urban-rural differences, Family planning policy

## Abstract

**Background:**

From 1982 to 2010, the country’s crude death rate (CDR) dropped sharply, fluctuated, and finally slightly declined. There is a big difference in CDR between urban and rural areas. From 1982 to 1990, the CDR in the country and the countryside declined, and the CDR in cities and towns rose. After 1990, the CDR in cities gradually decreased, the CDR in towns first fell and then rose, and the CDR in the countryside steadily increased. The CDR is affected by changes in the age-specific death rate (ASDR) and age structure.

**Methods:**

This paper decomposes CDR changes into the influence of declines in ASDR and the impact of age structure changes based on 1982, 1990, 2000, and 2010 census data.

**Results:**

The decline in ASDR reduces the CDR, and the aging population increases the CDR (including cities, towns, and the countryside). At the same time, decomposing the difference between the countryside and cities (or the countryside and towns) CDRs found that after 1990, the influence of ASDR differences and age structure differences increased with time. Our results revealed a more significant effect of ASDR differences. The combined effect of two factors (ASDR and age structure) makes the 0, 1–14, 15–64 age groups reduce the CDR, and the 65+ age group increases the CDR. In addition, the 0-year-old group has a not negligible impact on the changes in CDR, although it accounts for a small proportion of the total population.

**Conclusions:**

The influence of ASDR and age structure differs over time (1982 to 1990, 1990 to 2000, and 2000 to 2010) and across regions (cities, towns, the countryside). Considering the slow decline in ASDR and the accelerated aging population, we can infer that the CDR in 2020 will stabilize or even rise slightly instead of dropping significantly (compared with the CDR in 2010). This study provides a basis for the formulation of relevant public health policies.

## Background

The crude death rate (CDR) is defined as the number of deaths in a year per 1000 of the midyear population, and it is the simplest and most common measure of mortality [1]. CDR reflects the population’s health status and can be applied for the design and evaluation of health policies. Further, the rate of natural increase (in the absence of migration), measuring how quickly a population is growing or declining, is calculated by subtracting the CDR from the crude birth rate. In addition, these three indicators (CDR, crude birth rate, rate of natural increase) can help us figure out a country’s stage within the Demographic Transition Model. The three points mentioned above illustrate the importance of CDR.

The CDR is called “crude” because the denominator of the indicator includes people of all ages, but the death rate varies by age. In other words, the CDR is a function of age-specific death rate (ASDR) and age structure. In essence, a population with a high proportion of older persons will have a higher CDR than a population consisting of predominantly young persons. For example, due to the aging population in more developed countries, the proportion of older adults is comparatively high, and its CDR is about 11 deaths per 1000 population [2]. On the contrary, the CDR of less developed and least developed countries with relatively young people is only 7 deaths per 1000 population [2]. Therefore, a direct comparison of the CDR in two periods or populations may lead to misleading conclusions. As a result, it is vital to analyze how ASDR and age structure affects the change in CDR, which can be achieved by the decomposition method.

China is the most populous country in the world and even small population changes can cause widespread concern. According to the world population data table, China’s population would account for 18.04% of the world’s total population in 2021 [2]. Based on the above two points: 1) CDR is an important indicator and easily affected by age structure; 2) China has a huge population. Studying the trends and causes of the CDR changes in the Chinese context is essential.

The gap between urban and rural areas in China is still huge, although urban and rural areas have experienced unprecedented development since the reform and opening-up in 1978. Many scholars have studied the urban-rural gap from multiple angles, including income [3–7], education [8–12], health care [13], and housing wealth [14]. It should be noted that since 1982, the Chinese population census has divided the entire population into three categories: city population (*chengshi renkou*), town population (*zhen renkou*), and the countryside population (*xiangcun renkou*); the first two live in urban areas, and the third live in rural areas [15–18]. In other words, the urban population is the sum of the city population and town population, and the rural population equals the countryside population. Based on the above analysis, it is necessary to separately discuss the CDR in cities, towns, and the countryside and the differences between the three.

The risk of death varies by age. Population with many young children or population with a high proportion of older adults will have a relatively higher CDR because mortality risk increases at a very young age and the most senior age. It is common in demography to split the population into three broad age groups: children and young adolescents (under 15 years old), the working-age population (15–64 years), and the elderly population (65 years and older). Since the mortality at age 0 dropped significantly from 1982 to 2010 (from 35.22 per 1000 live births to 13.21), we divide the 0–14 year old group into two groups: 0-year-old group and 1–14 year old group. Therefore, it is necessary to analyze CDR changes by four age groups (0, 1–14, 15–64, 65+).

In this article, we developed a method of decomposing the change of CDR into contributions of ASDR changes and age structure changes. This paper proposes to use 1982, 1990, 2000, and 2010 census data to explore three questions: 1) From 1982 to 2010, the impact of the decline in ASDR and age structure changes on the CDR (including cities, towns, and the countryside); 2) Effects of ASDR and age structure on the countryside-city and the countryside-town CDR differences in 1982, 1990, 2000, and 2010; 3) From 1982 to 2010, the influence of 0, 1–14, 15–64, 65+ age groups on the changes of the CDR (including cities, towns, and the countryside). The study provides a perspective to better understand the changes in CDR in China. In the last section, we discuss the policy implications of our findings.

## Methods

Drawing on the decomposition method employed by Kitagawa [19], Das Gupta [20], and Canudas Romo [21], this article decomposes the change in CDR into two parts: the influence of age structure changes and ASDR changes. In addition, Saikia and Choudhury [22] have used this method to decompose the difference in CDRs in India from 1971 to 2011. Our study differs from theirs in that we focus on differences in CDRs across periods, regions, and ages in China. In contrast, Saikia and Choudhury focused on differences in CDRs across gender and states in India and compared their results with those of the USA and Sri Lanka.

Let the superscripts *x* and *y* denote different points in time, *i* represents i years old (*i* = 0, 1, ⋯, 89, 90+). The decomposition of CDR changes can be indicated as follows:

$$\Delta ={CDR}^y-{CDR}^x=\sum \limits_i{C}_i^y\cdot {M}_i^y-\sum \limits_i{C}_i^x\cdot {M}_i^x$$

The terms ${M}_i^x$ and ${M}_i^y$ represent the ASDR of *i* years old at times *x* and *y*. The terms ${C}_i^x$ and ${C}_i^y$ describe the proportion of *i* years old at times *x* and *y*. Δ can be decomposed into:

$${\displaystyle \begin{array}{c}\Delta =\sum \limits_i\left({C}_i^y-{C}_i^x\right)\times \left[\frac{M_i^x+{M}_i^y}{2}\right]+\sum \limits_i\left({M}_i^y-{M}_i^x\right)\times \left[\frac{C_i^x+{C}_i^y}{2}\right]\\ {}=\mathrm{the}\ \mathrm{influence}\ \mathrm{of}\ \mathrm{age}\ \mathrm{structure}\ \mathrm{differences}\\ {}+\mathrm{the}\ \mathrm{influence}\ \mathrm{of}\ \mathrm{ASDR}\ \mathrm{differences}\end{array}}$$

This factor decomposition method can analyze CDR changes in different periods in the same region and compare the CDR between two areas in the same period.

## Data

### Data sources

China’s death data come from five different sources: (1) population census, conducted in 1982, 1990, 2000, 2010, and 2020 by the National Bureau of Statistics of China (NBS). Death data include the numbers of deaths by age and gender across the country, cities, towns, and the countryside; (2) 1% national population sample survey, conducted in 1987, 1995, 2005, and 2015 by the NBS. Death data include the numbers of deaths by age and gender in the country, cities, towns, and the countryside; (3) 1‰ national sample survey on population changes, conducted every year except for population census years and 1% national population sample survey years by the NBS (from 1983 to present, except 1987, 1990, 1995, 2000, 2005, 2010, 2015, 2020). Death data include the numbers of deaths by age and gender in the country, cities, towns, and the countryside; (4) maternal and child health surveillance of China [23], conducted every year since 1991 by the National Health Commission of China (known as the Ministry of Health of China until 2013). Death data include the neonatal mortality rate, infant mortality rate, the under-five mortality rate, the maternal mortality rate in urban and rural areas; (5) United Nations inter-agency group for child mortality estimation [24]. Death data include infant mortality rate, mortality among children aged 1–4 years, and under-five mortality rate since 1969.

#### Data selection

To summarize, the characteristics of “large sample size”, “comprehensive data information” make population census data an ideal data source for this article. In addition, two critical points need to be clarified with data selection. First, before 1982, the usable data on mortality were CDR [25, 26], infant mortality rate, mortality among children aged 1–4 years, and under-five mortality rate. The 1982 census collected detailed death data for the year prior to the census for the first time [27]. Therefore, the 1982 census provided the basis for the in-depth development of mortality studies. Second, China carried out its seventh population census in 2020, but it is expected to take two years to compile the complete data (not the major data). Thus, on the premise that the 2020 population census data is not published in full, this article uses death data from the 1982, 1990, 2000, and 2010 census data to study the CDR.

The quality of death data in the census has always been controversial, and it is necessary to judge its reliability and accuracy. Scholars believe that the death data in 1990 was significantly lower than the actual value, especially the infant mortality rate [28]. On the contrary, the death data in 2000 was higher than the actual value [29]. Only the death data in 1982 are high quality and can be used directly [25, 30]. Although the death data in 1990 and 2000 have certain deficiencies, the census data is a relatively good choice in the absence of other data. Therefore, this study still uses the unadjusted death data in 1990 and 2000 for calculation. Moreover, the infant mortality rate in 2010 was lower than the actual value [31], so we adjusted it based on the 2010 Maternal and child health surveillance (13.10 per 1000 live births) [32].

## Results

### Decomposition by time

**Fig. 1:**
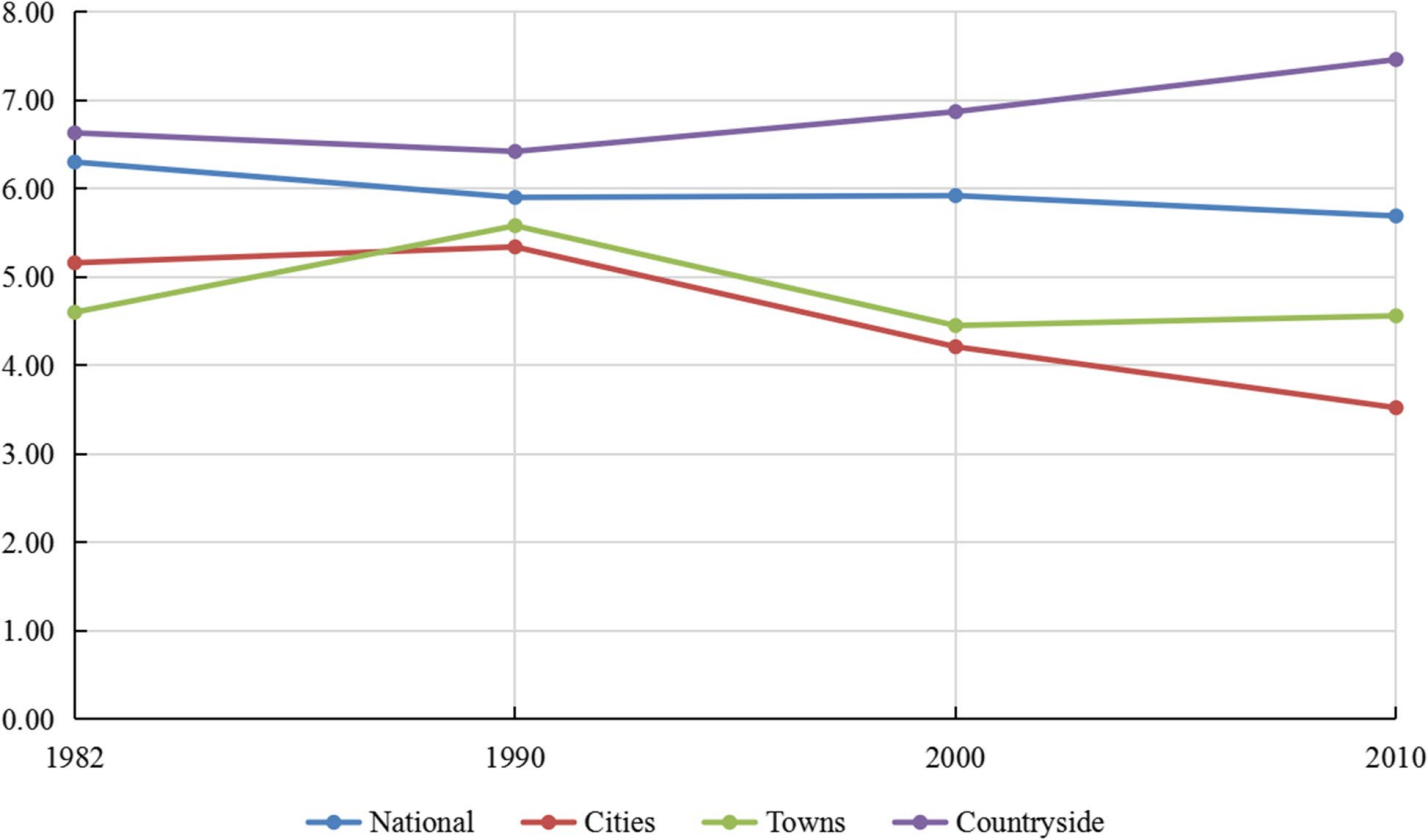
Crude death rate across the country, cities, towns and the countryside in 1982, 1990, 2000, and 2010. Data Sources: Calculated based on 1982, 1990, 2000, and 2010 census data [33–36]

Figure 1 shows the CDR across the country, cities, towns, and the countryside in 1982, 1990, 2000, and 2010. From 1982 to 2010, the country’s CDR dropped sharply, fluctuated, and finally slightly declined (1982: 6.30; 1990: 5.90; 2000: 5.92; 2010: 5.69 per 1000 population). There are significant differences in CDRs among cities, towns, and the countryside. From 1982 to 1990, the CDR in the country and the countryside declined, while the CDR in cities and towns rose. And then, after 1990, the CDR in cities gradually decreased (1990: 5.34; 2000: 4.21; 2010: 3.52 per 1000 population), the CDR in towns first fell and then rose (1990: 5.58; 2000: 4.45; 2010: 4.56 per 1000 population), and the CDR in the countryside steadily increased (1990: 6.42; 2000: 6.87; 2010: 7.46 per 1000 population).

**Fig. 2:**
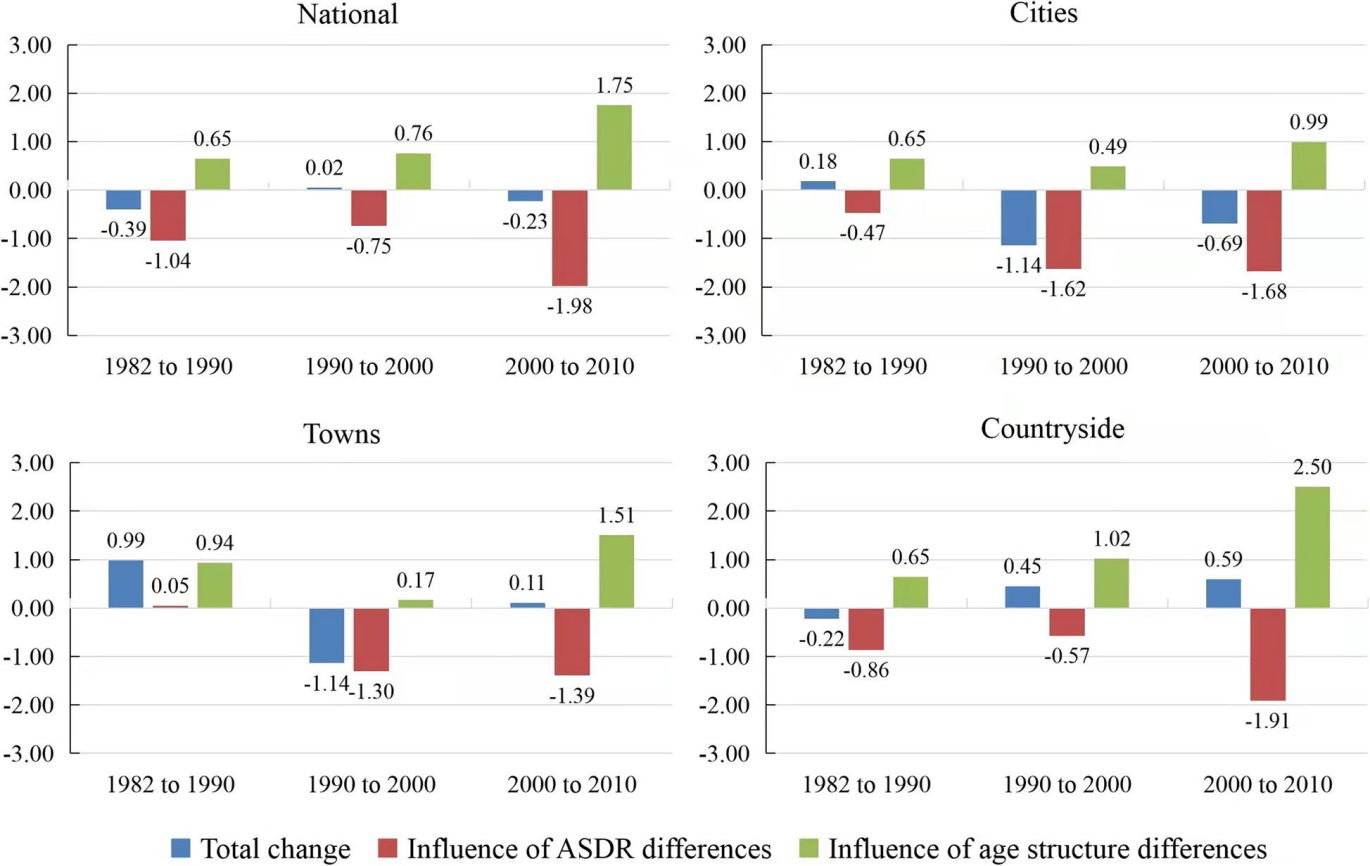
Decomposition of the crude death rates across the country, cities, towns, and the countryside from 1982 to 2010. Data Sources: Calculated based on 1982, 1990, 2000, and 2010 census data [33–36]

Figure 2 illustrates the decomposition results of the changes in CDRs across the country, cities, towns, and the countryside. Overall, the decline in ASDR has led to a reduction in CDR, but older age structure (also known as population aging) has increased CDR. According to the findings, the CDR will drop if the impact of ASDR declines is more significant than the impact of older age structure. The CDR will increase if the effect of ASDR declines is less than the influence of older age structure. In addition, for the three periods (1982 to 1990, 1990 to 2000, 2000 to 2010), the impact of changes in ASDR and age structure across the country in the third period is greater than the first two time periods. The increase is mainly attributable to a rapid fall in the ASDR and the accelerated population aging.

For towns, the impact of ASDR changes was just 0.05 per thousand points from 1982 to 1990. This result is primarily due to an increase in the ASDR of some people and a decrease in the ASDR of others. The effects of the ASDR of different age populations cancel each other out. For example, the ASDR of the 7-year-old group fell from 0.68 per 1000 population in 1982 to 0.63 in 1990, whereas the ASDR of the 70-year-old group increased from 39.50 per 1000 population in 1982 to 42.13 in 1990. During the same period, the influence of age structure changes in towns was 0.94 per thousand points. This result is mainly attributable to the increase in the proportion of the 0-year-old group (0.56 percentage points) and 65+ age group (1.19 percentage points) in the total population.

Looking at Fig. 2, the impact of the ASDR differences in cities increased significantly in the second and third periods (1990 to 2000, 2000 to 2010). For instance, the effect of the ASDR differences in cities is − 0.47 (1982 to 1990), − 1.62 (1990 to 2000), and − 1.68 (2000 to 2010) per thousand points in the three periods. Towns have a similar phenomenon. This result reflects the rapid decline in ASDRs between 1990 and 2010 in cities and towns. The fundamental reason is the switch-over from the centrally planned economy to the socialist market economy in China, after which China’s economy has flourished.

Similarly, the influence of changes in the ASDR in the countryside from 2000 to 2010 (− 1.91 per thousand points) has increased significantly compared to the first two time periods (1982 to 1990: − 0.86 per thousand points; 1990 to 2000: − 0.57 per thousand points). This result reflects that the ASDR in the countryside has dropped as rapidly as in cities and towns after 2000. The decline in the ASDR in the countryside is due to the gradual establishment of the new rural cooperative medical system after 2003 [37–39]. It solved the problem of expensive (and challenging) medical care for the countryside population. As a result, the ASDR in the countryside dropped rapidly.

**Fig. 3:**
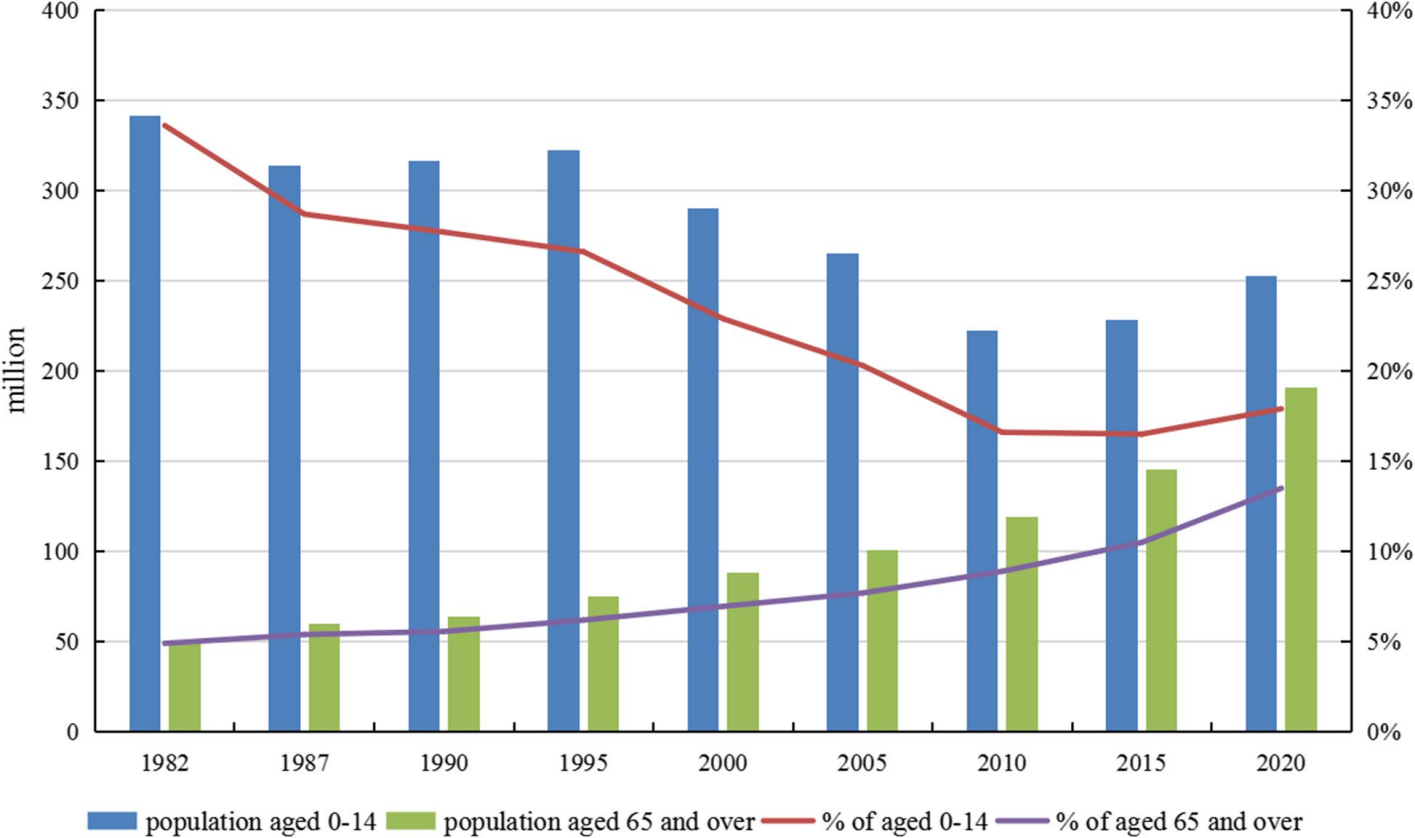
Population and proportions aged 0–14, 65+ in China from 1982 to 2020. Data Sources: China Statistical Yearbook (2021) [40]

In addition, Fig. 2 also shows that the nationwide influence of the age structure changes from 2000 to 2010 has increased significantly compared with the previous two periods (1982 to 1990, 1990 to 2000). This phenomenon reflects the accelerated population aging from 2000 to 2010. Figure 3 illustrates this point well. According to Fig. 3, the proportion of people aged 65 accounted for 8.90% of the total in 2010, increasing 1.94 percentage points from 2000 (1982 to 1990: 0.66 percentage points, 1990 to 2000: 1.39 percentage points) [40].

### Decomposition by region

**Fig. 4:**
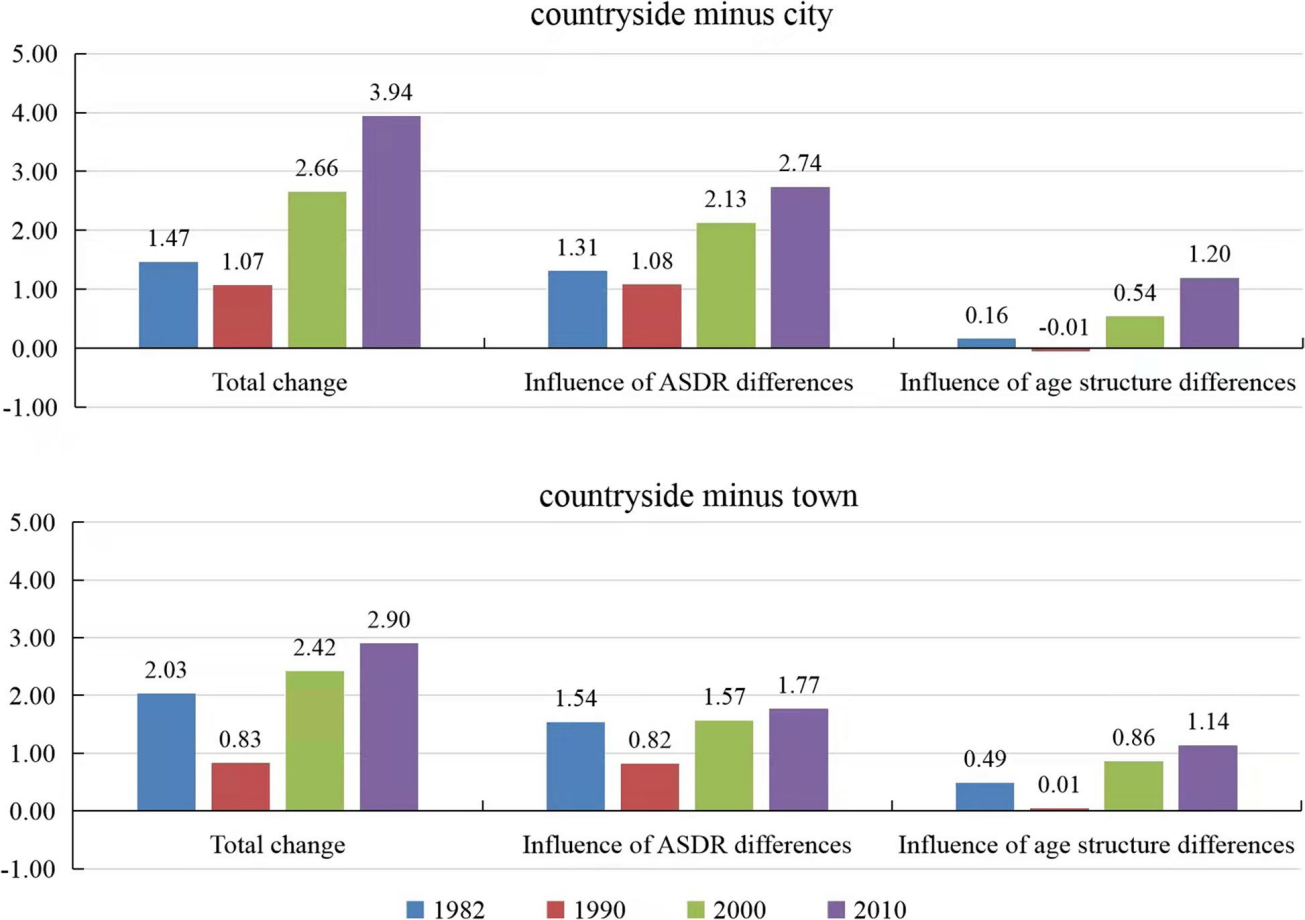
Decomposition of countryside-city, countryside-town CDR differences in 1982, 1990, 2000, 2010. Data sources: Calculated based on 1982, 1990, 2000, and 2010 census data [33–36]

Figure 4 lists the decomposition results of the countryside-city, the countryside-town CDR differences in 1982, 1990, 2000, and 2010. We can observe from Fig. 4 that from 1982 to 1990, the influence of ASDR differences and the effect of age structure differences decreased. After 1990, the influence of ASDR differences and the effect of age structure differences both increased over time. In addition, the impact of ASDR differences is greater than that of age structure differences.

In 1982, the countryside-city CDR difference was 1.47 per thousand points, mainly because the ASDR in the countryside is higher than that in the cities (the influence of ASDR differences is 1.31 per thousand points). Similarly, the differences in countryside-city and countryside-town CDRs in 1990, 2000, and 2010 were also caused by similar reasons.

After 1990, the influence of ASDR differences gradually increased in countryside-city and countryside-town. Moreover, the effect of the countryside-city ASDR differences in 1990, 2000, and 2010 (1.08, 2.13, 2.74 per thousand points) is greater than that in countryside-town (0.82, 1.57, 1.77 per thousand points). In addition, from 1990 to 2000, the growth rate of the influence of countryside-city ASDR differences (1.05 per thousand points, 97.22%) was greater than in countryside-town (0.75 per thousand points, 91.46%). This phenomenon reflects a gradual increase in ASDR differences between cities and towns. Similarly, the influence of countryside-city (− 0.01, 0.54, 1.20 per thousand points) and countryside-town (0.01, 0.86, 1.14 per thousand points) age structure differences have gradually increased in 1990, 2000, and 2010.

### Decomposition by age

Table 1 illustrates the age decomposition of the CDR changes across the country, cities, towns, and the countryside. Overall, the table shows that the decline in ASDR and changes in the age structure of 0, 1–14, and 15–64 age groups decreased CDR together (except to individual data points). Furthermore, the decline in ASDR and change in the age structure of the 65+ age group collectively increased the CDR. Additionally, the ASDR changes for all age groups have reduced the CDR. In contrast, the change in the age structure of the 15–64 and 65+ age groups increased the CDR. The role that the age groups play varies with times and regions.

**Table 1:** Decomposition of the country, cities, towns, and the countryside CDR changes by age

Area	Period	Total	Influence of 0-year-old group			Influence of 1–14 age group			Influence of 15–64 age group			Influence of 65+ age group		
			Subtotal	Influence of ASDR differences	Influence of age structure differences	Subtotal	Influence of ASDR differences	Influence of age structure differences	Subtotal	Influence of ASDR differences	Influence of age structure differences	Subtotal	Influence of ASDR differences	Influence of age structure differences
National	1982 to 1990	−0.39	− 0.21	− 0.26	**0.05**	− 0.26	− 0.24	− 0.02	−0.06	− 0.23	0.17	0.14	−0.31	0.45
	1990 to 2000	0.02	−0.20	0.07	**−0.27**	−0.14	− 0.07	−0.07	− 0.20	−0.45	0.25	0.55	−0.30	0.85
	2000 to 2010	−0.23	**−0.12**	− 0.15	**0.03**	**− 0.09**	−0.07	− 0.02	**−0.28**	− 0.66	0.38	**0.26**	−1.10	1.36
cities	1982 to 1990	0.18	−0.02	−0.07	0.04	−0.06	− 0.06	0.01	0.03	−0.07	0.10	0.23	−0.27	0.50
	1990 to 2000	−1.14	−0.23	−0.10	− 0.13	−0.13	− 0.08	−0.05	− 0.61	−0.78	0.17	−0.18	− 0.67	0.49
	2000 to 2010	−0.69	−0.01	− 0.01	0.00	− 0.04	−0.03	− 0.01	−0.44	− 0.63	0.19	− 0.20	−1.01	0.82
towns	1982 to 1990	0.99	0.07	−0.04	0.11	0.03	−0.01	0.03	0.26	0.16	0.10	0.63	−0.07	0.70
	1990 to 2000	−1.14	−0.24	−0.03	− 0.21	−0.17	− 0.10	−0.06	− 0.48	−0.68	0.20	−0.25	− 0.49	0.24
	2000 to 2010	0.11	−0.07	−0.08	0.01	−0.05	− 0.04	−0.01	− 0.21	−0.53	0.33	0.44	−0.74	1.18
countryside	1982 to 1990	−0.22	−0.17	− 0.24	0.07	− 0.26	−0.24	− 0.02	0.02	− 0.15	0.16	0.19	−0.24	0.43
	1990 to 2000	0.45	−0.24	0.12	−0.36	−0.17	− 0.08	−0.09	− 0.06	−0.37	0.31	0.93	−0.23	1.15
	2000 to 2010	0.59	−0.14	−0.23	0.09	−0.11	− 0.08	−0.02	0.00	−0.53	0.52	0.84	−1.08	1.92

Data sources: Calculated based on 1982, 1990, 2000, and 2010 census data [33–36]

The age groups play different roles in various times (1982 to 1990, 1990 to 2000, 2000 to 2010) and regions (the whole country and cities, towns, the countryside). For example, the national CDR changes from 1982 to 1990 were mainly affected by the age group of 1–14 years old. In addition, comparing the effects of different age groups (0, 1–14, 15–64, 65+) on the change in CDR, the 0-year-old group has a  not negligible influence (1982 to 1990: − 0.21 per thousand points; 1990 to 2000: − 0.20; 2000 to 2010: − 0.12).

A feature that needs to be clarified is the influence of the change in the age structure of the 0-year-old group across the country in the three periods (1982 to 1990, 1990 to 2000, and 2000 to 2010) were 0.05, − 0.27, and 0.03 per thousand points, respectively. It shows that the change in age structure during the second period (1990 to 2000) is greater than that in the other two periods. This characteristic exists in the whole country, cities, towns, and the countryside. How to understand this feature? This is mainly due to the change in the proportion of the 0-year-old population. Taking national data as an example, the proportion of the 0-year-old population *increased slightly* from 1982 to 1990 (0.16 percentage points) and from 2000 to 2010 (0.16 percentage points), but it *dropped significantly* from 1990 to 2000 (1.08 percentage points).

## Conclusions and Discussion

Since the 1980s, China’s CDR has experienced a process of sharp decline, slight fluctuations, and minor declines. Based on the census data in 1982, 1990, 2000, and 2010, this paper uses a decomposition method to decompose CDR changes into the influence of ASDR changes and age structure changes. The main conclusions of this study are as follows:

First, the decline in ASDR reduces the CDR, and the aging population increases the CDR (including cities, towns, and the countryside). The reason is that the rapid development of society and the economy has caused a rapid decline in ASDR [41–43]. Data show that China’s per capita GDP had increased from 533 yuan in 1982 to 30,808 yuan in 2010 (72,000 yuan in 2020) [40]. The aging population is caused by many factors: 1) The strict family planning policy (the one-child policy) implemented from 1980 to 2007 and the rapid decline in fertility desire caused a sharp drop in the total fertility rate (from 5.80 in 1970 to 1.18 in 2010) [44–46]. 2) Improving living standards, medical standards, and hygiene standards have increased the average life expectancy.

Second, decomposing the difference between the countryside and cities (or the countryside and towns) CDRs found that after 1990, the influence of ASDR differences and age structure differences increased with time. Comparing the two revealed a more significant effect of ASDR differences. The following reasons could explain this. On the one hand, the ASDR in the countryside is higher than that in cities and towns, and the decline rate is slower than that in cities and towns. Because per capita income, medical conditions, and participation rate of basic medical insurance in the countryside are lower than those in cities and towns. On the other hand, since the 1980s, the number of migrant workers has increased, and the growth rate has accelerated. According to the 2020 Migrant Workers Monitoring Survey Report, the average age of migrant workers is 36.6 years old, of which 66.8% are 40 years old and below, and 14.2% are over 50 years old [47]. The continuous and large-scale rural-to-urban migration has promoted the younger population of cities and towns (and the aging of the countryside population). Specifically, the scale of migrant workers in 1989, 2001, and 2010 were 30.00 million, 89.61 million, 153.35 million, respectively [48–50].

Third, the combined effect of two factors (ASDR and age structure) makes the 0, 1–14, 15–64 age groups reduce the CDR, and the 65+ age group increases the CDR. For the 65+ age group, the proportion of the total population increased, and the ASDR decreased. Still, the former had more effect than the latter, so the 65+ age group increased the CDR. In addition, the 0-year-old group has a not negligible impact on the CDR changes, although it accounts for a small proportion of the total population (1982: 1.94%; 1990: 2.11%; 2000: 1.02%;2010: 1.19%). First, the implementation of the one-child policy and the decline of fertility desire have significantly reduced the proportion of the 0-year-old group (from 1.94% in 1982 to 1.19% in 2010). Second, in recent decades, the rapid economic development, the improvement of living standards, and the continuous improvement of medical services and sanitation conditions have all contributed to a substantial reduction in mortality (especially mortality at age 0) [from 35.22 per 1000 live births in 1982 to 13.21 in 2010]. Considering the above two reasons, the 0-year-old group has played a vital role in changing the CDR.

**Fig. 5:**
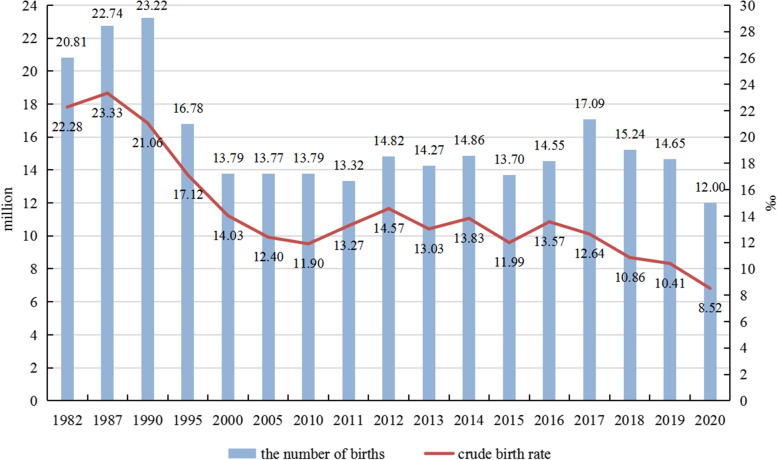
Birth rate and the number of births in China from 1982 to 2000. Data sources: The birth rate is from China Statistical Yearbook (2021) [40]. The number of births: 1) Data for 1982, 1990, 2000, and 2010 are from population census data [33–36]; 2) Data for 1987, 1995, 2005, and 2015 are from 1% national population sample survey data [55–58]; 3) Data for 2011 to 2019 are from China Population and Employment Statistical Yearbook (2012–2020) [59–67]; 4) Data for 2020 comes from the Statistical Bulletin of China’s Health Development (2020) [68]

Fourth, the CDR in 2020 will not drop significantly but will stabilize or even rise slightly (compared with the CDR in 2010). On the one hand, the population aging will be more evident in 2020. There are two reasons: 1) the number of births has not increased significantly after 2010. Since 1980, the Chinese government has successively implemented the following family planning policies: the one-child policy (1980), the policy that allows couples to have a second child if each parent is an only child (2007) [except Henan province, which followed in 2011] [51], the policy that allows couples to have a second child if one of the parents is an only child (2013), the universal two-child policy (2015), and the three-child policy (2021). According to Fig. 5, the crude birth rate rose slightly from 1982 to 1987, fell sharply from 1987 to 2010, fluctuated around 13 births per 1000 population from 2011 to 2016, and dropped rapidly from 2017 to 2020 (of which, it fell to a new low of 8.52 births per 1000 population in 2020). From another perspective, China’s fertility rate did not continue to rise after 2007, although the government gradually relaxed its family planning policy in 2007, 2013, 2015, and 2021. Scholars generally believe that China’s fertility rate will not rise sharply in the future (more likely to fluctuate or even decline) [15]. The number of births in China in Fig. 5 confirms our conjecture. From 2005 to 2016, the annual number of births fluctuated from 13.5 to 15.0 million. The number of births increased to 17.09 million in 2017 and has declined since then (a record low in 2020: 12 million). 2) The number and proportion of older adults aged 65 and above are increasing. With the improvement of living standards, the average life expectancy in China has increased from 74.83 years to 77.30 years (2010 to 2019) [40, 52]. In addition, from 2010 to 2020, the number of people aged 65 and over has increased from 118.94 million to 190.64 million; the proportion of people aged 65 and over has increased from 8.9% to 13.5% [40]. In short, the decline in the number of births and the increase in the number of people aged 65 and over have made the population aging even more severe. On the other hand, the mortality of centenarians has not decreased noticeably in recent decades, but a significant mortality decline in younger age groups [53]. In other words, the average life span can grow due to medical and societal advancement, but maximal lifespan, even in the greatest of circumstances, is eventually restricted by biology: the very last surviving people live to 120 years old, not 150 or 200 [54]. Based on the above analysis (slow decline in ASDR and accelerated aging population process), it can be inferred that the CDR in 2020 will stabilize or even rise slightly instead of dropping significantly.

This study provides a basis for the formulation of relevant public health policies. The CDR is affected by age distribution. The relatively older population generally will have higher CDR, even if two regions have the same ASDRs. From 1982 to 2010, the changing trends of the CDR in cities, towns, and the countryside were different. We analyzed the reasons behind them. The increase in the CDR in the countryside after 1990 was mainly due to the accelerated population aging. Its essence is that the countryside’ economic development and medical care are lower than in cities. Therefore, public health policies should be formulated with particular preferences for the countryside. In addition, the government should develop policies to focus on solving the social security problems of left-behind elderly (especially living alone) and disabled elderly in the countryside.

The study also has certain limitations. One limitation is that the quality of the census data influences our results and conclusions. In analyzing the urban-rural differences in China’s CDR changes, we have no access to the 2020 population census, which is, of course, more representative in describing the recent changes. However, the decomposition of China’s CDR is still meaningful. The results and conclusions can help deepen the understanding of China’s CDR changes and their causes.

## Data Availability

The data described in this article can be freely and openly accessed on the website of the National Bureau of Statistics of China: http://www.stats.gov.cn/tjsj/pcsj/

## Abbreviations

ASDR: Age-specific death rate
CDR: Crude death rate
NBS: National Bureau of Statistics of China
PCO: Population Census Office under the State Council
